# The elusive relationship between retinal anatomy and brain amyloid

**DOI:** 10.1093/braincomms/fcaf038

**Published:** 2025-02-19

**Authors:** Dilraj S Grewal, Sharon Fekrat

**Affiliations:** iMIND Study Group, Department of Ophthalmology, Duke University School of Medicine, Durham, NC 27710, USA; iMIND Study Group, Department of Ophthalmology, Duke University School of Medicine, Durham, NC 27710, USA

## Abstract

This scientific commentary refers to ‘Retinal microstructure and microvasculature in association with brain amyloid burden’, by Egle *et al*. (https://doi.org/10.1093/braincomms/fcaf013).

**This scientific commentary refers to ‘Retinal microstructure and microvasculature in association with brain amyloid burden’, by Egle *et al*. (https://doi.org/10.1093/braincomms/fcaf013).**

Pathology of β-amyloid remains a key feature in the pathogenesis of Alzheimer’s disease and is a focus area of several emerging therapeutics. β-Amyloid burden may be preceded, often by years, by alterations in cerebrovascular regulation, and there is keen interest in assessing the retinal structure and microvasculature as a more easily accessible surrogate than neuroimaging.

Egle *et al*.^1^ assess the association of retinal microstructural and microvascular signs with cortical amyloid burden in the prospective Atherosclerosis Risk in Communities study. The authors are to be commended for undertaking this important work to evaluate the association of retinal measures with amyloid burden.

Using participants without a diagnosis of dementia from the Atherosclerosis Risk in Communities study, the authors evaluated participants with neuropsychological testing, brain magnetic resonance imaging, positron emission tomography scans and optical coherence tomography (OCT) and OCT angiography (OCTA) imaging using the RTVue-XR Avanti SD-OCT (Visionix, Fremont, CA, USA) system. They investigated 121 participants without a diagnosis of dementia who underwent florbetapir positron emission tomography between 2011 and 2013 and then 4–8 years later, OCT and OCTA imaging. Intermediate capillary plexus vessel density was associated with increased continuous amyloid standardized uptake value ratio.

While the work is promising, in the current sample, the OCTA metrics were not sufficiently powered as predictors to detect a significant effect with continuous amyloid standardized uptake value ratio. OCTA images were evaluated using the 3 × 3 mm OCTA images in 53 participants, and while the smaller scan area has a higher sampling rate and scan density than the larger 6 × 6 mm area, it is possible that measuring changes over a larger area of the macula, extending further beyond the foveal avascular zone, may be helpful to better evaluate these very subtle changes.

Larger retinal vascular changes assessed on fundus photographs have been shown to be associated with cognitive decline and dementia. Specifically, it has been shown that retinopathy is associated with accelerated rates of 20-year cognitive decline^2^ as well as with increased continuous β-amyloid burden, above and beyond vascular risk factors in adults without dementia.^3^ The authors statistically accounted for covariates including diabetes and hypertension, which are known to cause reduction in vessel density on OCTA and reduction in macular thickness on OCT, even in the absence of clinically detectable retinopathy, and these remain potential confounders for the wider application of retinal imaging. A future goal remains trying to differentiate microvascular changes associated with systemic vascular factors like diabetes and hypertension from those associated with Alzheimer’s disease. They also appropriately adjusted for age, sex, race, APOE e4 status and axial length, all of which impact the OCT and OCTA quantitative measures and need to be accounted for when considering deployment of retinal imaging for population-based screening.

OCTA is particularly suspectable to scan quality artefact, which is demonstrated by having only 53 (50%) participants with high-quality OCTA images available for study.^4^ In contrast, there were 117 participants that had OCT retinal nerve fiber layer thickness measurements of adequate quality, which is consistent OCT being a more robust modality in terms of scan quality compared with OCTA. This also suggests that when conducting such prospective studies involving different retinal and neuroimaging modalities, it may be worthwhile to obtain OCTA imaging first and then proceed with the more expensive and invasive positron emission tomography and magnetic resonance imaging only if high-quality OCTA imaging was obtained. As the OCTA technology advances with higher axial and lateral resolutions and faster scan acquisition times, it may become feasible to capture high-quality images more consistently across a larger area of the macula, particularly in individuals with dementia who often have difficulty maintaining fixation. These developments would help better characterize the retinal findings in this population.

A key unmet need in OCTA research in neurodegenerative diseases remains lack of standardization of reporting metrics. Various metrics such as vessel density, vessel length density and perfusion density, among others, have been reported so far. The segmentation slabs used to define superficial, intermediate and deep capillary plexuses vary among different device manufacturers and can also vary across different software versions on the same device. In addition, the thresholding algorithms used to calculate vessel density are different across devices making cross study comparisons impossible unless they are conducted using the same device, software and scan acquisition protocol. This remains a limitation in testing retinal measures in larger more diverse populations, which will be critical for validation of retinal biomarkers. It is challenging to conduct large studies with neuroimaging and retinal imaging concurrently and then follow study participants longitudinally as evidenced by the observation that many participants in this study underwent OCTA imaging years after their positron emission tomography imaging. Prospective studies are often unable to take advantage of the advances in imaging technology as all longitudinal imaging is usually locked into the baseline protocol. Age-related decline in OCT and OCTA measures is accelerated in individuals with neurodegenerative diseases, and this may be difficult to control for when there is a large temporal separation between neuroimaging and retinal imaging.^5^ Concurrently captured and then longitudinally followed data are critical to better understand the relationship between retinal architecture, retinal microvasculature and amyloid deposition.

Improvements in software technology to handle different data inputs across devices and create a standardized output would be very desirable. Similarly, conversion factors to compare across devices and gender such as those available for macular thickness on OCT are also required for OCTA. It is conceivable that a study population focused on those with high amyloid burden may be more enriched to detect retinal changes and there may be a certain threshold of amyloid burden before there are detectable alterations in retinal structure or microvasculature.

A pathophysiological basis for only the intermediate capillary plexus to have a significantly associated vessel density remains unclear. The authors recognize that this may be due to the statistical weakness of using a dichotomized amyloid measure. The authors did not find an association between ganglion cell layer and amyloid burden. Our iMIND group’s prior work has shown the ganglion cell layer (often measured as a ganglion cell-inner plexiform layer complex due to difficulty in accurately segmenting the two layers) is a high-performing biomarker, even in early neurodegenerative processes.^6^ Assessment of retinal amyloid as a tool for diagnosis of Alzheimer’s disease is currently under investigation by several groups using hyperspectral imaging, some of which have received fast-track designation from the US Food and Drug Administration underscoring the importance of this approach.^7^ In addition, the ability of using retinal metrics to assess retinal changes over time due to amyloid is also of increasing importance given the emergence of therapies to reduce amyloid burden. While the clinical meaningfulness of anti-amyloid monoclonal antibody trial results is still debated, the promise of valid longitudinal retinal measures that correlate with cerebral amyloid remains enticing.^8^

The study underscores the importance of a convergence of improved OCTA technology with better scan quality and consistent definitions of segmentation layers and measurement metrics to further our understanding of the relationship between retinal and cerebral changes in neurodegenerative diseases. As our hardware advances, coupled with improvements in software analytics, we continue to make meaningful interdisciplinary progress towards valid and more accessible retinal imaging endpoints.
